# Mapping whole-organism genetic comorbidities across model Species using unified ontologies

**DOI:** 10.1093/genetics/iyag038

**Published:** 2026-02-10

**Authors:** Caitlin Peaslee, Madison Held, Eloise Fadial, Donald F Conrad

**Affiliations:** Center for Embryonic Cell & Gene Therapy, Oregon Health & Science University, 3303 S. Bond Ave, #CH13N, Portland, OR 97239, United States; Division of Genetics, Oregon National Primate Research Center, Oregon Health and Science University, 505 NW 185th Ave, Beaverton, OR 97006, United States; Division of Genetics, Oregon National Primate Research Center, Oregon Health and Science University, 505 NW 185th Ave, Beaverton, OR 97006, United States; Center for Embryonic Cell & Gene Therapy, Oregon Health & Science University, 3303 S. Bond Ave, #CH13N, Portland, OR 97239, United States; Division of Genetics, Oregon National Primate Research Center, Oregon Health and Science University, 505 NW 185th Ave, Beaverton, OR 97006, United States

**Keywords:** *H. sapiens*, *M. musculus*, *D. rerio*, *D. melanogaster*, *C. elegans*, human, mouse, zebrafish, fruit fly, C. elegans, spermatogenesis, sperm, male infertility, infertility, comorbidities, genetics, phenotypes, genotype-phenotype, MGI, FlyBase, WormBase, HPO, ZFin

## Abstract

Understanding how genetic variation contributes to organism-wide phenotypes is critical for identifying mechanisms of disease. Here, we present a computational approach to analyze whole-organism comorbidities associated with genes underlying non-obstructive azoospermia (NOA), the most severe form of male infertility. We curated 204 mouse genes with experimentally validated spermatogenic failure and mapped their orthologs and phenotype annotations across humans, *M. musculus*, *D. rerio*, *D. melanogaster,* and *C. elegans* using a unified cross-species phenotype structure. This framework integrates a newly developed reproductive phenotype ontology with standardized whole-body phenotype categories to enable direct cross-species comparisons, using thousands of genotype-phenotype associations stored in model organism databases. Our analysis shows that most NOA genes have conserved orthologs across models, and that perturbation of these genes is frequently associated with non-reproductive phenotypes. In particular, integumentary defects and neoplastic phenotypes recur across species, supporting a shared genetic basis for epidemiological links between male infertility and systemic disease. Gene-level comorbidity patterns are partially predictable from single-cell RNA-seq, whole-body gene expression, and Gene Ontology annotations, suggesting that fundamental biological constraints shape the systemic consequences of reproductive gene dysfunction. Clustering genes by comorbidity profiles further distinguished genes with isolated reproductive effects from those with broad organismal consequences. This work demonstrates the power of ontology-based cross-species analysis for identifying pleiotropic effects of Mendelian disease mutations, and provides a resource, CoMorbidity DataBase Mapper (CoMo DBM), for joint analysis of genotype-phenotype associations in model organism databases.

## Teaser

Men with genetic causes of non-obstructive azoospermia (NOA) are more likely to have co-occurring diseases, like cancers, cardiovascular disease, and diabetes, and shorter lifespans than fertile men. Peaslee et al. used known model organism genetic models of NOA to understand the genetic connections between NOA and other disease traits. The authors looked across evolutionarily-distant species to determine the extent of the conservation of these genotype-phenotype connections in humans, mice, zebrafish, fruit flies, and roundworms by constructing a computational pipeline for querying and analyzing data from model organism databases. They found that some comorbidities of genetic infertility are recurrent across species, including integumentary and neoplastic phenotypes.

## Introduction

Epidemiological studies have provided strong evidence that male infertility is interconnected with overall health (Ventimiglia et al. 2015; Choy and Eisenberg 2018; Kimmins et al. 2024). Large cohort studies have shown that men with low sperm counts are at increased risk of comorbidities, including hypertension, hyperlipidemia, cardiovascular disease, hyperuricemia, skin disorders, and cancers relative to the general population (Abdel-Naser and Zouboulis 2016; Eisenberg et al. 2016; Boeri et al. 2022). Among cancers, lymphoma, melanoma, and testicular, prostate, and pancreatic cancers have been specifically isolated as associated with male infertility (Tarín et al. 2015; Hanson et al. 2016; Ramsay et al. 2024). These data clearly indicate that an infertility diagnosis in apparently cancer-free men is a risk factor for future cancer. For instance, in a study of over 76,000 infertile men and 760,000 control men from a health claims database covering the years 2001 to 2009, survival analysis of infertile men showed a higher incidence of all cancers and individual cancers compared to controls (Eisenberg et al. 2015). Similarly, male infertility can be a biomarker of cancer for first- and second-degree relatives of men with azoospermia (Hanson et al. 2018).

Despite these associations, the mechanistic basis of these comorbidities remains unclear. An estimated 20% of idiopathic male infertility cases are caused by large-effect Mendelian mutations in one of hundreds of genes (Nagirnaja et al. 2022). A reasonable hypothesis is that some infertility-related comorbidities stem from shared genetic mechanisms. For example, defects in some DNA repair genes confer risk for infertility and cancer (Nagirnaja et al. 2018). Testing this hypothesis in humans is challenging due to incomplete knowledge of the genetics; the phenotyping of these patients is limited and typically restricted to reproductive traits, and the follow-up of adult patients with a genetic diagnosis is laborious. These effects are further amplified by the condition's locus heterogeneity, which slows the collection of case series.

**Fig. 1.**
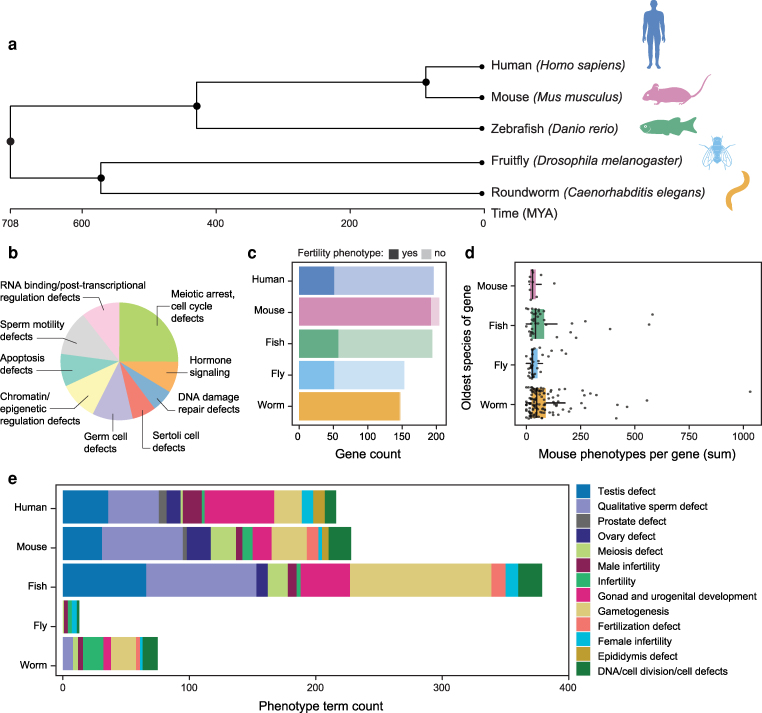
a) A phytogenic tree displays the evolutionary relationships between model organisms created by TimeTree, annotated with species-specific traits (Kumar et al. 2017). b) A summary of defects of mouse model studies of the mouse *NOA genes* published in the MGI, with the chart allowing multiple annotations per gene. c) Chart displaying the total orthologs of the mouse *NOA genes* in humans, zebrafish, fruit flies, and roundworms with light bars. Dark bars annotate the count of *NOA genes* having at least one phenotype in the categories Abnormality of the Genitourinary System or Fertility per species. d) Mouse *NOA genes* are annotated by the oldest species with a high-confidence ortholog, plotted as mouse phenotypes per gene for each oldest species. e) The count of phenotype ontology terms per species in the category *Fertility*, collected from HPO, MGI, Zfin, FlyBase, and WormBase.

Model organism studies offer tremendous value in investigating the genetic basis of infertility and its comorbidities. Gene-targeting efforts have systematically mutated or silenced nearly all protein-coding genes in *C. elegans*, *D. melanogaster,* and *M. musculus*, generating comprehensive phenotyping datasets. It is increasingly common to generate comprehensive phenotype data on mutants, which are described using standardized phenotype ontologies and are recorded in databases. We hypothesized that genes that cause non-obstructive azoospermia (NOA), the most severe form of male infertility, frequently act in fundamental cellular pathways (e.g. DNA repair, chromatin remodeling, cell-cycle control) that are used in other organ systems. As a result, perturbation of these genes may lead to **predictable comorbidity profiles** that can be detected across multiple model organisms when orthologous genes are disrupted. Here, we leverage model organism databases to assess whether any infertility-related comorbidities can be observed repeatedly across organisms upon orthologous gene perturbation. Our analysis spans phyla Chordata (*H. sapien*s, *M. musculus*, and *D. rerio*), Arthropoda (*D. melanogaster*), and Nematoda (*C. elegans*) (Fig. 1a). These metazoan species share conserved transcriptional programs in reproductive tissues, reflecting evolutionary conservation of sexual reproduction and gametogenesis (Ramm et al. 2014; Brattig-Correia et al. 2024).

Historically, the major model organism communities have developed and used their own species-specific phenotype ontologies to describe experimental results, and most genetic knowledge within each species is encoded in this way. To connect and compare these rich genotype-phenotype datasets across organisms, the genetics community has started to build species-agnostic or species-neutral ontologies. The Monarch Initiative is a major consortium driving this effort and has played a central role in establishing infrastructure for cross-species analysis (McMurry et al. 2016). The Monarch platform integrates multiple species-agnostic ontologies, including Uberon for anatomy (Mungall et al. 2012), MONDO for disease (Vasilevsky et al. 2025), and uPheno for phenotype harmonization (Matentzoglu et al. 2025), and provides interactive tools for exploring genotype-phenotype relationships across model organisms and humans. The Alliance of Genome Resources is another important effort, explicitly working with species-specific communities to build tools for cross-species data integration and analysis (Bult and Sternberg 2023).

While these resources are seeking to address the most general case of unifying knowledge across all species, they are complex and evolving, and our study required a simpler framework with additional constraints. First, we wanted a simple cross-species ontology defined for our five organisms of interest that could be used to quantify whole-organism comorbidity patterns associated with a defined set of genes. Second, we required a stable cross-species phenotype structure that could be held constant through the development of the project. Third, reproductive phenotypes- particularly those related to male reproduction and spermatogenesis- remain coarse in existing species-agnostic ontologies, motivating the development of a more targeted ontology for this biological domain. Fourth, we wanted a cross-species ontology that would be computationally straightforward and simple to query and analyze for the model organism databases of interest, potentially in a highly iterative manner as we developed our study.

To perform this analysis, we therefore systematically aggregated and compared genotype-phenotype associations across the five species, using a two-part cross-species phenotype ontology. The first component is a specialized reproductive phenotype ontology that we developed for this study, designed to match homologous cell types, molecular processes, and reproductive defects across species. This was paired with a broader “whole-body” phenotype categorization provided by the Model Organisms Aggregated Resources for Rare Variant ExpLoration (MARRVEL) (Wang et al. 2017), which aggregates phenotype annotations from multiple model organism databases into standardized categories. Using this combined framework, we curated a database of mouse genes with experimentally validated spermatogenic failure phenotypes from the Mouse Genome Informatics (MGI) database and analyzed orthologous genes and phenotypes in humans, zebrafish, fruit flies, and roundworm. We interpreted patterns of comorbidities using scRNA-seq data from the testis, full-body tissue gene expression, and gene ontology annotations. We identified genes with consistent comorbidities across multiple species, as well as some comorbidities that appeared to be enriched, as a class, with infertility gene perturbation more broadly. This work highlights opportunities and bottlenecks in the emerging field of multi-species genetic analysis, and the potential for integrating model organism databases for understanding comorbidities in human genetic disease.

## Materials and methods

### Data acquisition

#### Mouse spermatogenic failure/azoospermia genes

Genes associated with spermatogenic failure and azoospermia in mice were compiled from MGI (Baldarelli et al. 2024) by manually searching genes associated with azoospermia-related phenotype terms. We excluded less severe forms of male infertility than azoospermia, such as oligozoospermia. We manually reviewed publications for each gene and recorded metadata on testis histology, infertility traits, and comorbidity descriptions where available. The NOA gene list contains 204 unique mouse genes, excluding multi-genic genotypes and incomplete spermatogenic failure (Supplementary Table 3). The mouse *NOA genes* were broadly characterized by their mouse genotypes, summary gene ontology terms, and gene function databases. The genes were manually broadly summarized by (1) meiotic arrest and cell cycle defects, (2) DNA damage and repair defective, (3) sperm motility defective, (4) defective Sertoli cells, (5) RNA binding and post-transcriptional regulation defects, (6) hormone signaling defects, (7) defective germ cells, (8) apoptosis defective, and (9) chromatin and epigenetic regulation defects, allowing multiple summary categories per gene (Fig. 1b).

#### NOA gene ortholog prediction and filtering

We converted these mouse genes to orthologs in humans, zebrafish, fruit flies, and roundworms by g:Profiler (Kolberg et al. 2020). High-quality gene orthologs were filtered by including only high-confidence predicted orthologs with the “best” DIOPT prediction score (Hu et al. 2011; Wang et al. 2017; Kolberg et al. 2023).

#### Assigning each gene ortholog an “oldest species.”

Each mouse *NOA gene* was assigned the *oldest species* label based on the most distant last common ancestor first, then to the most recent last common ancestor in the following order: roundworm, fruit fly, zebrafish, and then mouse. Then, we plotted the number of mouse phenotypes per gene, categorized by their oldest species label.

#### Phenotype ontology terms for reproduction-related traits

We searched species-specific phenotype ontology term databases for all terms impacting reproduction from HPO, MGI, FlyBase, ZFin, and WormBase phenotype ontology trees (Gargano et al. 2024; Larkin et al. 2021; Ruzicka 2015; Harris 2020). We mapped the species-specific phenotype ontology terms into the 19th abnormal phenotype category, “Abornomality of Reproduction” (Gargano et al. 2024; Sternberg et al. 2024; “Mouse Genome Database (MGD)”). The reproduction category is further broken down into 13 reproduction-related groups.

#### The CoMo DBM pipeline accesses the MARRVEL API for genotype-phenotype data

The CoMorbidity DataBase Mapper (CoMo DBM) pipeline in R Studio reads input gene lists and ontology terms for each species into the workspace environment. CoMo DBM accesses the MARRVEL API, which integrates 21 databases to assess gene and variant function and integrate data between model organisms, using R Studio (Wang et al. 2017). Gene symbols can be read in by any of the species and converted to other orthologs by “gconvert”, which returns NCBI Entrez IDs based on NCBI RefSeq IDs for the target species (Kolberg et al. 2020). CoMo DBM begins with input gene symbols, converts the symbols to Ensembl gene IDs, and then MARRVEL returns JSON-formatted metadata. Phenotype data is parsed from the JSON file, and each phenotype for each species with appropriate homology is counted and added to a final table.

The phenotype data returned from MARRVEL includes species-specific phenotype ontology codes, as well as a cross-species ontology created by the MARRVEL developers that they named “MCat.” There are 18 MCat categories: Prenatal Development or Birth; Growth Abnormality; Nervous System; Eye and Ear; Integument; Head and Neck; Limb/appendage; Skeletal and Connective Tissue; Blood and Immune; Cardiovascular; Musculature; Respiratory; Digestive; Genitourinary; Endocrine; Metabolism; Neoplasm; Other. To implement our additional “Fertility” MCat category, the fertility terms for each species are searched and added to a nineteenth MCat category after being counted. The *Fertility* MCat describes reproduction and spermatogenesis more precisely than the MCat “genitourinary” term, which roughly corresponds to the HPO group of terms under *Abnormality of the genitourinary system*: HP:0000119. The final output is a table with gene symbols and homologous genes for each species, Entrez IDs, diopt score, confidence level, and a count of phenotypes in each MCat category for each gene in each species.

### Statistical analysis

#### Significance testing on phenotypes per gene in biological categories

To assess the association of NOA gene mutation and disease in each species, we tabulated phenotype counts for each MCat category, comparing the proportion of NOA gene orthologs with the phenotype to the proportion of all mouse orthologs with the phenotype. We used the Fisher exact test to test for an association, and then performed multiple-test correction on the full set of resulting *P*-values (i.e. from across all species) by controlling the false discovery rate at q < 0.05. Significance was determined with an α-significance level of 0.05.

#### Model organism genome gene list downloads

The full genome gene lists were downloaded for each model organism from the NIH NCBI Datasets database at https://www.ncbi.nlm.nih.gov/datasets/.

The reference versions for each species are as follows:

- *H. sapiens*: Taxonomy ID 9606; assembly GRCh38.p14; GenBank GCA_GCA_000001405.29; RefSeq GCF_000001405.40
- *M. musculus*: Taxonomy ID 10090; assembly GRm39; GenBank GCA_000001635.9; RefSeq GCF_000001635.27; Strain C57BL/6J
- *D. rerio*: Taxonomy ID 7955; assembly CRCz11; GenBank GCA_000002035.4; RefSeq GCF_000002035.6; Tuebigen Strain
- *D. melanogaster*: Taxonomy ID 7227; Release 6 plus ISO1 MT; GenBank GCA_000001215.4; RefSeq GCF_000001215.4
- *C. elegans*: Taxonomy ID 6239; WBcel235 assembly GenBank GCA_000002985.3; RefSeq GCF_000002985.6; Bristol N2 Strain

#### Correlations calculations

To calculate the correlations between the human and mouse phenotypes per gene, we filtered those genes with at least one phenotype in any MCat. We calculated a complete pairwise correlation coefficient between the phenotypes associated with mouse and human genes. The correlation calculations between the sum of phenotypes between humans and mice were performed with the R::Correlation, Variance, and Covariance package (“R: Correlation, Variance and Covariance”) (Matrices). A complete pairwise correlation for each gene was calculated using the Pearson method. NA values propagated from incomplete observations where the standard deviation was zero, or missing values from missing orthologs were replaced with r = 0. Observations were plotted with ggplot::geom_point() and ggExtra::ggMarginal() to add a marginal histogram (Wickham 2016; “ggMarginal function—RDocumentation”). The axes were transformed to a logarithmic scale with ggplot::log_x_scale_log10() and log_y_scale_log10().

#### Clustering

NOA genes were clustered by k-means clustering with R::kmeans using the default Hartigan and Wong (1979) algorithm. We determined the number of clusters after raising k until redundant clusters were observed. To formally test the goodness of clustering, we calculated a gap statistic with R::cluster “clusGap()”, We tested from k = 1 to *k* = 20 with 100 bootstrap samples, *B* = 100, and nstart = 25. After k = 4 each additional cluster was not significantly different in its features from the existing clusters.

#### Multinomial regression

Using the “nnet” package in R Studio, we fit multinomial logistic regression models to the clustered gene sets (Ripley 2023). Gene expression was log-transformed before analysis. To assess the goodness of fit of these models, we used a pseudo-R^2^ measure attributed to McFadden, calculated as 1—[ln(L_model_)/ln(L_0_)] (McFadden 1974; Ripley 2023). A *P*-value for model fit was calculated by the likelihood ratio test.

## Results

### A cross-species reproductive phenotype ontology

To assess reproductive phenotypes from aggregate databases, we manually standardized infertility-related terms from each organism database (HPO, MGI, FlyBase, ZFin, and WormBase), creating a cross-species phenotype ontology (Supplementary Table 1). Each database hosts a different phenotype ontology tree, so we manually searched and categorized the reproductive phenotype codes of each species into 13 broad groups, including DNA/cell division defects, meiosis, gametogenesis, gonad/urogenital development, quantitative sperm defect, testis function, fertilization, and general male or female infertility (Methods). Phenotype ontology terms were similar among vertebrates, though zebrafish had 75% more reproductive phenotype terms than humans or mice (Fig. 1e). Notably, the “gametogenesis” category contained 112 terms in zebrafish, compared to 22 in humans and 28 in mice. Invertebrates had fewer reproductive phenotype terms overall: fruit flies had only 13, while roundworms had 74. A key distinction was that fruit fly reproductive terms described general fertility defects without detailed terms for sperm quantity, sperm quality, or fertilization. The only fruit fly term specifically describing gametogenesis is *meiotic cell cycle defective: FBcv:0000431*.

### Integration with the MARRVEL ontology

A key feature of MARRVEL is its cross-species phenotype categories, which the creators term “MCat” (Methods, Supplementary Table 2) (Wang et al. 2017). There are 18 MCat categories curated in MARRVEL. These include abnormalities of prenatal development or birth, growth, the nervous system, eyes and ears, integument, head or neck, musculoskeletal system, blood and blood-forming tissues, the cardiovascular system, the musculature, the respiratory system, the digestive system, the endocrine system, metabolism or homeostasis, and “other” categories including the breast, cellular phenotypes, the voice, the thoracic cavity, and constitutional symptoms (Supplementary Table 2). Species-specific differences in reproductive phenotype ontologies present challenges for cross-species genotype-phenotype comparisons, so we have collected all reproduction-related terms into one new MCat group created for this study: “fertility.” This new *Fertility* MCat category describes reproduction and spermatogenesis more precisely than the MCat category “genitourinary.”

### A computational pipeline for tabulating genotype-phenotype associations across species

Spermatogenesis is an essential and highly conserved process across sexually reproducing species (Box 1) (Ramm et al. 2014; Murat et al. 2023). To systematically compare NOA-associated phenotypes across model organisms, we developed the CoMorbidity DataBase Mapper (CoMo DBM), a computational pipeline that integrates reproductive and non-reproductive genotype-phenotype associations from five model organisms. We compiled and manually curated 204 genes linked to monogenic spermatogenic failure and NOA from mouse studies reported in the MGI database for input into CoMo DBM (Method, Supplementary Table 3). We broadly grouped these genes into nine mechanistic categories, including (1) meiotic arrest and cell cycle defects, (2) DNA damage and repair defects, (3) sperm motility defects, (4) RNA binding and post-transcriptional regulation defects, (5) hormonal signaling defects, (6) defective germ cells, (7) apoptosis defects, (8) chromatin and epigenetic regulation defects (Fig. 1b, Method, Supplementary Table 4). To evaluate spermatogenic failure across model organisms, we identified orthologs of the 204 mouse NOA genes in zebrafish, fruit flies, and roundworms (Fig. 1c). We filtered gene orthologs by high- and moderate-confidence DIOPT prediction scores (Methods, Supplementary Table 5, Fig. 1c) (Hu et al. 2011). We traced each mouse *NOA gene* to its most evolutionarily distant species with a medium or high confidence ortholog, the “oldest species” (Methods, Supplementary Table 6). Most (61%) *NOA genes* were traced back to roundworms, 16% to fruit flies, 18% to zebrafish, and only 5% to mice (Method, Fig. 1d). Notably, this pattern of conservation was not significantly different from that of a random set of 1,800 human protein-coding genes (Supplementary Fig. 1). On the other hand, NOA genes have older recognizable orthologs than 761 genes involved in innate immunity (X^2^ = 76, *P* < 2.2 × 10^−16^), but younger orthologs than genes associated with human neural degeneration (Human Phenotype Ontology identifier HP:0002180) (X^2^ = 13.2, *P* < 0.005). We hypothesized that genes with older orthologs might be involved in more biological processes and thus might have more comorbidities. We found no significant difference in the number of mouse phenotypes associated with NOA gene perturbation and the age of the oldest ortholog of each gene.

Box 1. Introduction to spermatogenesis When spermatogenesis is functional in humans, germ cells in the seminiferous tubules of the testis undergo a series of asymmetric mitotic divisions during spermiogenesis. One daughter cell maintains the stem cell pool (Type A spermatogonia) while the other daughter cell migrates toward the lumen of the seminiferous tubule (Type B spermatogonia) (L’Hernault 2006). The Type B spermatogonia will then proceed into meiosis I and II to create four round spermatids. These spermatids then enter spermiogenesis, where their chromosomes become tightly packed with nuclear and transition proteins that will later be packed around protamines, the head of the cell becomes capped with an acrosome, and a centriole elongates to form the sperm tail (Couteau and Zetka 2011). The resulting spermatozoa are flagellated cells ready to leave the seminiferous tubes and enter the epididymis for maturation.

Developing sperm cells rely on support cells for nutrients, cell clearance by apoptosis, chromatin packing, immune functions, and more. Sertoli cells are essential cells that (1) help transport developing sperm cells toward the lumen of the seminiferous tubule, (2) pass nutrients from the bloodstream through the blood-testis barrier (BTB), (3) secrete androgen binding proteins, (4) produce the glycoproteins anti-Müllerian hormone (AMH) and Inhibin B, (5) interact with tubular Leygid cells for autocrine and paracrine regulation, (6) support phagocytosis of residual bodies during spermiogenesis (L’Hernault 2006; Gunes et al. 2015; Iliadou et al. 2015). Leydig cells produce androgens, bind with luteinizing hormone to stimulate cAMP production to supply cholesterol to the mitochondria, and signal in the testis and throughout the body via paracrine and autocrine signaling (Iliadou et al. 2015; Zirkin and Papadopoulos 2018). The ratios of support cells to germ cells are tightly regulated by apoptosis; removal of defective cells allows for maintaining sperm quality and hormonal regulation (Shukla et al. 2012). In addition, gonadotropins (human chorionic gonadotropin (hCH) and follicle-stimulating hormone (FSH)) released from the hypothalamus of the brain are required for stimulating testosterone production for supporting spermatogenesis (Xie et al. 2022). The control of the hypothalamic-pituitary-gonadal (HPG) axis is critical in regulating support cells, spermatogenesis, and reproductive traits (Xie et al. 2022). These critical pathways that allow germ cells to differentiate into sperm and then travel outside of the reproductive tract can break, leading to infertility and co-occurring diseases.

### Testing for comorbidities of genetic NOA in aggregate

With CoMo DBM, we counted the number of MCat phenotypes for each NOA ortholog, separately in each species (Methods, Supplementary Table 6). We found that 192/204 (94%) of NOA genes are linked to a fertility phenotype in mice using the CoMo DBM pipeline. As all genes in our list are known to cause a mouse infertility phenotype, this indicates that there is a modest false negative rate when detecting association with the CoMo DBM approach. The percent of NOA gene orthologs linked to a fertility defect is 42% in humans, 27% in zebrafish, 99% in roundworm, and 32% in fruit fly (Fig. 1c).

**Fig. 2.**
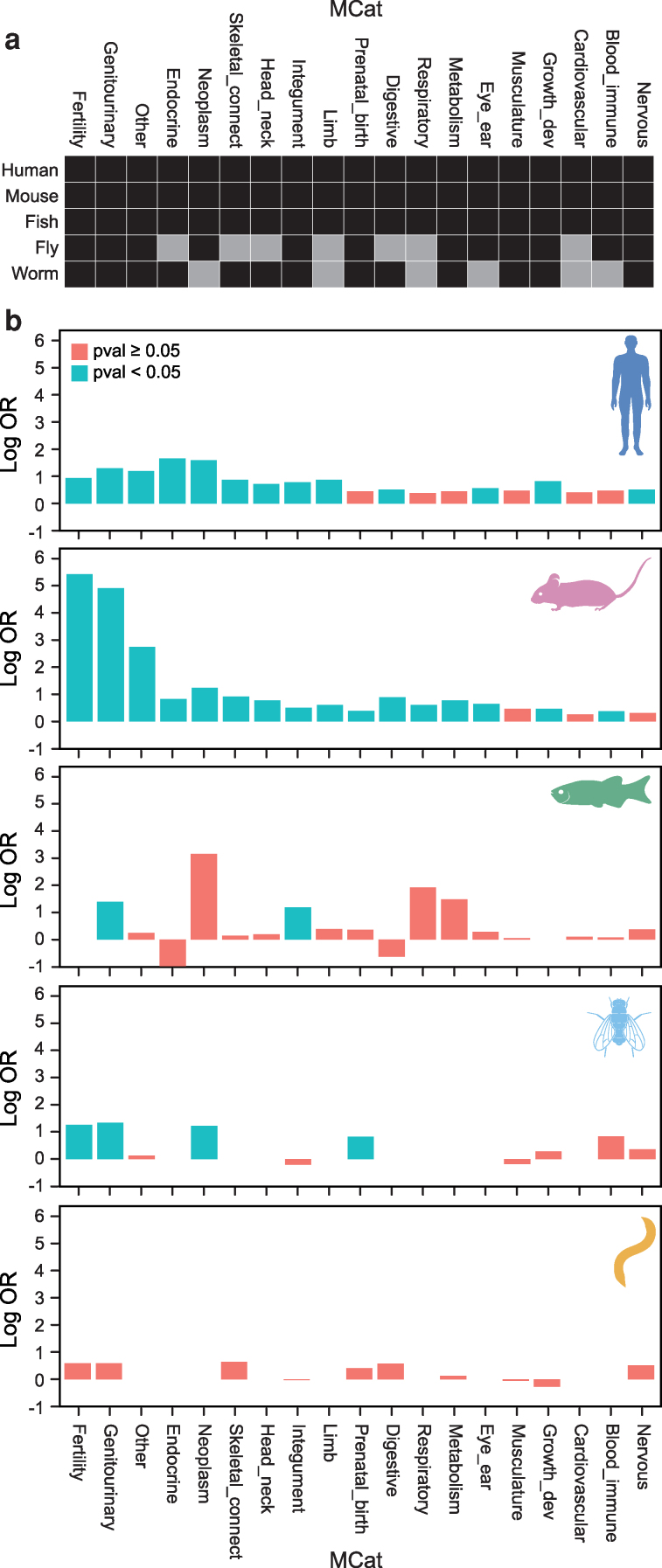
a) The phenotyping map of MCat categories across the five species considered here. Black squares reflect categories that are phenotyped, while grey squares represent categories that are not phenotyped/not relevant for a given species. For instance, the “eye & ear” trait category is not phenotyped in worm. b) The association between MCat phenotype counts and NOA gene perturbation across humans, mice, zebrafish, and fruit flies. The log odds ratio (OR) summarizes the odds of obtaining a phenotype in each category, compared to the full set of mouse orthologs in each species-specific protein-coding genome. We restrict the analysis in each species to genes with mouse orthologs, to control for any systematic differences that may exist based on this evolutionary ascertainment. *P*-values correspond to a Fisher Exact test of the hypothesis that the Log OR = 0. lLog odds for categories that are not phenotyped in a species have no bar for that species.

To formally test for an association between NOA genes and phenotypes in each MCat category, we performed Fisher's exact tests comparing NOA genes to the full genome in each species (i.e. all genes in the genome with a mouse ortholog; Methods, Fig. 2b, Supplementary Table 7). Mutations in *NOA genes* and their orthologs are significantly associated with phenotypes in fertility and abnormalities of the genitourinary system in mice (OR = 228, *P* < 1e-145 and OR = 136, *P* < 1e-114), humans (OR = 2.5, *P* < 1e-4 and OR = 3.6, *P* < 1e-10) and fruit flies (OR = 3.52, *P* < 2e-7 and OR = 3.8, *P* < 2e-8) (Fig. 2b). NOA genes were significantly associated to genitourinary defects in zebrafish (OR = 4.0, *P* < 2e-5), but we were unable to evaluate infertility as the MARRVEL API does not provide the underlying ZFIN phenotype codes. The only outlier was roundworm, whose orthologs were not associated with infertility or genitourinary defects (OR = 1.8, *P* = 1 and OR = 1.8, *P* = 1). Even though >90% of NOA orthologs are associated to a fertility-related trait in roundworms, the rate of fertility-related phenotypes for roundworm mutants in general is extremely high, as we discuss later, and thus our observed OR was not significant. Overall, these results support the conservation of the *NOA gene* functions in spermatogenesis across distant model organisms despite species-specific differences in phenotype term annotations.

### Non-reproductive *NOA gene* phenotypes

Non-reproductive phenotypes are significantly associated with *NOA gene* orthologs throughout model organisms (Supplementary Table 7, Fig. 2b). We observed an association across most phenotype categories in the mouse, and 10/16 non-reproductive categories in the human. In fish, only the integument was significantly associated (OR = 3.3, *P* < 1e-3), where 4% (9 of the 209) of NOA orthologs have an integument phenotype compared to 1.3% of all genes. In flies, both the neoplasm and prenatal/birth categories were significantly associated (OR = 3.4, *P* = 0.02 and OR = 2.3, *P* < 5e-3). However, this testing framework is meant to identify comorbidities that are consistently associated with NOA genes in a species; there are certainly other comorbidities of specific NOA genes that are not detected with this approach. We therefore explored recurrence of comorbidities at a single-gene level below.

### Class association: integumentary system

In humans, over 20 genetic disorders with co-occurring integumentary and reproductive defects have been reported (Abdel-Naser and Zouboulis 2016). The most common conditions are dyschromias (color alterations in skin, nails, or hair), ichthyosis (dry, scaly, or thickened skin), and genodermatoses (genetic skin disorders) associated with cryptorchidism and hypogonadism. These connected disorders between the integument and male reproduction can broadly arise from androgen signaling, germ cell dysregulation, and inflammation (Ceruti et al. 2018; Umaoka et al. 2020; Khandpur et al. 2021).

*NOA gene* orthologs are significantly associated with abnormalities of the integumentary system in humans, mice, and zebrafish. In zebrafish, NOA orthologs with reported phenotypes in integument include *Dicer1*, *Foxp3a*, *Kita*, *Kitlga*, *Lamin a/c*, *Nrg1*, *Pafah1b1a*, *Prdm1* and *Slc12a2*. Of these, five (*Dicer1, Foxp3a, Kita, Pafah1b1a,* and *Prdm1)* impact pigmentation, affecting melanocytes, stripe coloration, and chromatophores that produce and contain pigment. These genes are broadly involved in stem cell migration, proliferation, and differentiation; for example, KIT and PRDM1 play central roles in the specification and migration of primordial germ cells (PGCs) and neural crest-derived melanocytes (Kolberg et al. 2020). Both PGCs and neural crest-derived cells undergo extensive embryonic migration and rely on overlapping signaling and regulatory pathways. As a result, genetic perturbations affecting cell migration, survival, or differentiation frequently produce parallel phenotypes in germ cells and neural crest derivatives, including combined reproductive and pigmentation or neurodevelopmental defects. Our results suggest that zebrafish are underappreciated as a potential model for studying the comorbidity of male infertility and integumentary defects.

### Class association: neoplasm

Neoplasm is significantly associated with *NOA gene* orthologs in mice, humans, and fruit flies (Fig. 2b). Notably, in zebrafish, only 3/12,774 genes in the genome with mouse orthologs are associated with neoplasm as annotated by MARRVEL, and 1 of these, *gja1*/*cx43* is an NOA gene (OR = 23, *P* = n.s.). *NOA genes* in mice and humans that have phenotypes in both reproduction and neoplasm include *Ar/AR, Atm/ATM, Brca1/BRCA1, Brca2/BRCA2, Dicer1/DICER1, Fancf/FANCF, Fancm/FANCM, Kit/KIT, Mlh1/MLH1, Smad4/SMAD4,* and *Wt1/WT1*. Mouse *NOA genes* that have phenotypes both in reproduction and neoplasm but not in human orthologs include *Arid4b*, *Bax, Bcl2l1, Bcl2l11, Cdk1, Cdk2, Cdk4, Hmga2, Igf1r, Ikbkg, Kitl, Mad2l2, Mcm8, Msh5, Polg, Prdm1, Rnf8, Slx4, and Zbtb16* (Supplementary Table 6). Of these, *ARID4B, BAX, BCL2L1, BCL2L11, CDK1, CDK2, HMGA2, IGF1R, IKBKG, KITL, MSH5, PRDM1,* and *RNF8* are not annotated with human phenotypes in neoplasm or fertility from CoMo DBM output. These mouse “neoplasm + infertility” genes that have yet to be linked to human male infertility are good candidates for additional scrutiny during genetic analysis of infertility patients.

In the human *NOA gene* orthologs, 29 intersect with known cancer genes from the COSMIC database of human cancer gene mutations (Supplementary Table 8) (Tate et al. 2019). Of these, 11 genes have germline mutations that are tumor suppressors and oncogenes that are hallmarks of cancer, including *AR, ATM, BRCA1, BRCA2, CDK4, DICER1, FANCF, KIT, MLH1, SMAD4,* and *WT1*. Mutations in these genes are causative for somatic prostate, ovarian, breast, colorectal, endometrial, thyroid, and pancreatic cancers, leukemias, embryonal rhabdomyosarcoma, blastomas, central nervous system tumors, Wilms tumor, and desmoplastic small round cell tumor (Supplementary Table 8) (Tate et al. 2019). In summary, these results support a possible genetic contribution to the epidemiological association of male infertility and cancer, highlight possible animal models to study mechanistic connections between infertility and cancer, and nominate candidate genes that have yet to be linked to infertility and cancer in humans.

### Single-gene analysis of comorbidities

**Fig. 3.**
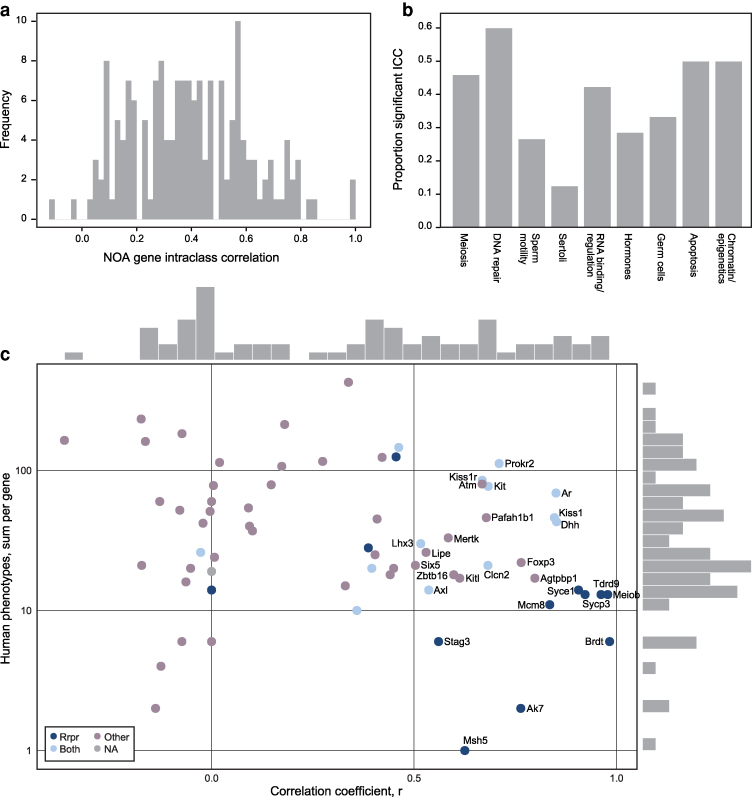
a) The distribution of phenotype intraclass correlation coefficients (ICCs) for NOA gene orthologs. Higher values reflect greater similarity in phenotype vectors among species. b) The proportion of genes with significant ICC values (*P* < 1e-3 via permutation test), stratified by functional annotation (as defined in Supplementary Table 4). c) The correlation coefficient for each gene was calculated by comparing phenotypes per MCat in mouse and human *NOA genes*. Human phenotypes per gene are plotted vs the correlation coefficient, and the fill color annotates OMIM disease categories. OMIM disease categories include “repro”: only reproductive diseases associated with the gene, “other”: only non-reproductive diseases associated with the gene, “both”: the gene is associated with both reproductive and non-reproductive diseases, and “NA”: the gene is not included in OMIM. Only genes with at least one human phenotype are included. The Y-axis is log10 transformed, and the X and Y marginal histograms correspond to frequencies of genes at each position.

Above, we tested for comorbidity categories that are associated with NOA gene perturbation, as a class, within each model organism. Even if there is no systematic association between NOA gene orthologs and a particular trait in a species, the species could still be a good model for studying the comorbidities of individual genes. As a complementary approach, we assessed for each single gene the extent of recurrence of comorbidities, using the intraclass correlation coefficient (ICC) of each gene's phenotype vector. This measures the similarity in phenotypes associated with a given gene across all species. The median ICC across NOA genes was 0.389 (max 1.0, Fig. 3a). Using permutation testing, we found 60 genes with ICC values greater than expected due to chance (*P* < 1e-3). When tabulated across our functional gene annotations (Supplementary Table 4), we saw that genes involved in basic cellular processes, shared across many cells and species, were more likely to have significant ICC values, while genes that function primarily in cell types that are not evolutionarily conserved have smaller ICC values (Fig. 3b). For instance, “DNA damage and repair” (60%), “Apoptosis” and “Chromatin/epigenetic regulation” have the highest rates of significant ICC values, while “Sertoli cell” (11%) and “Sperm motility” (25%) have the lowest.

### Human diseases associated with *NOA gene* orthologs

To evaluate the concordance between mouse and human phenotype annotations of the *NOA genes*, we calculated the correlation between the MCat phenotype vectors for human and mouse *NOA genes* (Fig. 3c, Supplementary Table 9) Remarkably, there are dozens of genes with 10–100 associated human phenotypes, that also have high phenotype correlation (R > 0.5) to their cognate mouse models. We further annotated genes with the diseases in OMIM for each gene and then by a summary OMIM disease annotation category(Amberger et al. 2019). The OMIM disease summary categories are “Reproductive Diseases Only,” “Both Reproductive and Non-Reproductive Diseases,” and “Non-Reproductive Diseases Only” (Fig. 3c, Supplementary Table 9).

Overall, the picture observed through this analysis matches what was observed looking at the single-gene intraclass correlations (which is a more general correlation analysis expanded to more species). Genes with the highest phenotype correlation between mouse and human tend to have more specialized function in the testis, have expression restricted to a single testicular cell type (Supplementary Table 10), and are less likely to have co-morbidities in humans. The OMIM diseases in this set were spermatogenic failure, premature ovarian failure, hypogonadotropic hypogonadism, and precocious puberty (Amberger et al. 2015). The genes associated with both reproductive and non-reproductive diseases tend to have broader expression patterns, more general cellular functions, but can still have reasonably high phenotype correlation to the mouse. The genes in this category include *WT1*, *AR*, *DHH*, *KIT*, *CLCN2*, and *LHX3*. *LHX3* has enriched expression in the brain, and the other five genes have enriched expression in Sertoli cells of the testis (Fagerberg et al. 2014; Mahyari et al. 2021). The OMIM diseases associated with the genes in this category broadly vary, including endocrine dysregulation disorders, cancers, developmental disorders, sex reversal syndromes, and neuropathies (Supplementary Table 9). The genes without a human NOA association may have many human phenotypes, but a poorer correlation with the mouse. Many of these genes are linked to severe human syndromes; we hypothesize that humans affected with these syndromes may have spermatogenic impairment, but are not phenotyped for testicular function due to their comorbid conditions. The genes in this category include *AGTPBP1, FOXP3, PAFAH1B1, ATM, KITLG, MERTK, LIPE,* and *SIX5,* and the OMIM disease associations in this category include developmental disorders, neurodegenerative disease, immune dysregulation, retinitis pigmentosa, cancers, and more, including multiple disorders with childhood-onset.

### Clustering of genes by comorbidity pattern

**Fig. 4.**
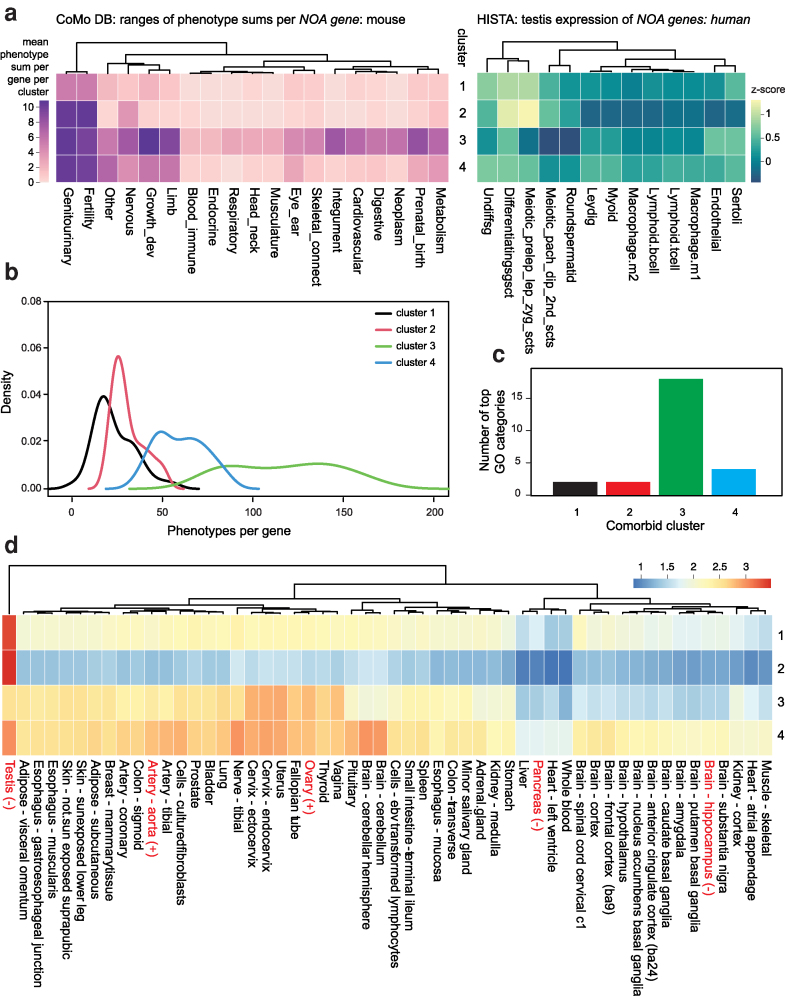
a) Left: the CoMo DBM output of mouse phenotypes per *NOA gene* per MCat, clustered by k-means clustering with k = 4. Right: average cell-specific gene expression *Z*-score for *NOA genes*, grouped by the same k-means clustering as the left panel. Gene expression is shown for 13 testicular cell types, as generated by the HISTA human scRNA-Seq atlas of testis gene expression. b) Distribution of the number of phenotypes per gene, for each of the four comorbidity clusters. c) The number of GO categories with maximum enrichment per cluster. We calculated the fraction of genes in each cluster with each GO annotation. Comorbidity cluster three had the highest proportion of annotated genes for 18/26 categories evaluated. d) Heatmap of the mean log transcripts per million (TPM) from GTEx bulk RNA-Seq, and genes are grouped by comorbidity clustering as in panel a. GTEx RNA-Seq data are generated from hundreds of human postmortem donors. Expression level in five tissues was significantly associated with increased (+) or decreased (−) frequency of comorbidity; these are indicated with red organ labels.

We clustered NOA genes by their mouse MCat phenotype counts, identifying four distinct groups by k-means clustering (Fig. 4a). NOA genes fell into four broad groups: 1) genes linked to isolated infertility, with few or no associated comorbidities (*n* = 42, 21%); 2) genes with extensive reproductive phenotypes, but also enriched for phenotypes in the “other” category (*n* = 84, 42%) 3) genes with broad comorbidities, specifically enriched for integument, neoplasm, and growth and development (*n* = 22, 11%) and 4) genes linked to nervous system defects, sometimes with a small number of other traits in growth and development, metabolism, eye and ear, and blood-immune categories (*n* = 43, 20%).

The primary difference between clusters one and two is the “other” MCat category traits. While 82% of genes in cluster two have an “other” annotation, only 59% in cluster one do. These tend to be molecular or cellular traits. For example, of the 69 genes with an “other” annotation in cluster two, at least 24 are involved in DNA repair or recombination, with mouse phenotype labels such as “Abnormal meiosis,” “Abnormal chiasmata formation,” “Increased cellular sensitivity to DNA-damaging agents,” etc. The other difference is in the extent of characterization: genes in cluster one have only 5.3 phenotypes/gene in the “genitourinary” category, roughly half the number in cluster two (10.3 phenotypes/gene). More generally, clusters three and four had more total phenotypes per gene compared to clusters one and two (mean of 117 and 60 phenotypes/gene vs 23 and 31, respectively, Fig. 4b).

### Dissecting drivers of comorbidity

Next, to better understand the biological and molecular basis of these comorbidity clusters, we attempted to model cluster membership as a function of testis single-cell gene expression, multi-tissue gene expression, and gene ontology (GO) annotations (Methods). For testis gene expression, we used a human testis single-cell RNA sequencing (scRNA-seq) atlas, “HISTA”(Mahyari et al. 2021, 2025). There are two major classes of cell types in HISTA germ cells: e.g. spermatogonia, spermatocytes undergoing meiosis, and spermatids; and somatic cells: Sertoli cells, Leydig cells, endothelial cells, and immune cells (M1 and M2 macrophages, B cells, and T cells). We calculated z-scores for gene expression across 13 testicular cell types in HISTA and filtered for *NOA gene* orthologs (Supplementary Table 10). We used multinomial logistic regression to model comorbid cluster membership as a function of scRNA-seq expression. In this model, HISTA scRNA-seq expression explained 22% of the variance between clusters (R^2^ = 0.22, log-likelihood = −115.5 model; −224.6 null, *P* = 4 × 10^−7^).

What are the drivers of this association? Among clusters one and two, four germ cell types show enriched expression: the two classes of spermatogonia, and the two classes of spermatocytes (Fig. 4a). Additionally, cluster one showed modest enrichment in Sertoli cells. Notably, postmeiotic germ cells show no enrichment in these clusters. In contrast, clusters three and four show much more enrichment in somatic cell types compared to germ cells. Cluster three gene expression is enriched in differentiating spermatogonia (median *z*-score 0.48), Sertoli cells (median *z*-score = 0.31) and endothelial cells (z = 0.23). Cluster four genes are enriched in 10 cell types (all median z > 0), seven of which are somatic. The top somatic cell classes are Leydig (3rd overall, median z = 0.39) and Sertoli (rank 4, median z = 0.37).

We expanded the analysis of gene expression to the whole human body by annotating human NOA genes with the GTEx bulk RNA tissue expression in non-diseased human donors (GTEx Consortium 2013). Non-diseased donors (also called “normal” donors) had no evidence of cancer, infectious disease, or inflammatory disease, ≥21 or ≤70 years of age, body mass index of ≥18.5 or ≤35, never been diagnosed of metastatic cancer, not received chemotherapy or radiation therapy for any condition within past two years, no history of intravenous drug abuse, and no history of HIV/AIDS/HCV/HBV exposure (Carithers et al. 2015). We filtered the full GTEx RNA-seq gene expression for human NOA orthologs across 54 tissues (Fig. 4d, Method, Supplementary Table 11). Fitting a multinomial logistic regression model with these data, GTEx tissue expression explained 52% of the variance between clusters (*R*^2^ = 0.52, log-likelihood = −110.5 model; −232.6 null, *P* = 8.3 × 10^−6^). Thus, the comorbidity pattern of an NOA gene is strongly influenced by its whole-body pattern of gene expression. To distill this analysis into single-tissue observations, we binarized the comorbidity clusters into two groups: those with some comorbidity in non-reproductive tissues (clusters three and four) and those with effects restricted to fertility (clusters one and two). We fit a simple logistic regression of binary comorbidity state to the GTEx expression data, and found that NOA gene expression in 15 tissues was associated with comorbidity. The expression level in three tissues was associated with a lower frequency of comorbidity; notably expression in testis (β=−3.9, *P* = 1 × 10^−4^), pancreas (β=−2.5, *P* = 0.01), and hippocampus (β=−2.0, *P* = 0.04). Conversely, comorbidity was positively associated with expression in two tissues, including ovary (β=2.9, *P* = 4 × 10^−3^), and the aorta (β=2.4, *P* = 0.02). These tissue labels are highlighted in red in Fig. 4d.

We gathered gene ontology annotations (GO) of molecular functions and biological processes for each mouse *NOA gene* from the MGI (Supplementary Table 12). Again, modeling comorbidity clustering with a multinomial log-likelihood model, we found that GO molecular function annotation was associated with cluster assignment (R^2^ = 36.4%, *P* = 4.5 × 10^−12^). We evaluated 26 GO categories with at least one gene annotated in this set. Interestingly, comorbidity clusters three and four, which are enriched for comorbidities compared to clusters one and two, show a higher rate of GO annotations per gene (cluster three = 10.9, cluster four = 7.3 vs cluster one = 4.6 and cluster two = 4.1). Comorbidity cluster three had the highest proportion of annotated genes for 18/26 categories (Fig. 4c). The categories “Cell differentiation” and “Cellular component organization” applied to 50 and 63% of genes in cluster one, but these labels were frequent in all four comorbidity clusters; no other GO category was labeled on a majority of genes in this cluster. Likewise for cluster two. On the other hand, 100% of genes in cluster three were annotated as “response to stimulus,” and 91% were labeled as “signaling”; other highly enriched categories included system development (77%), programmed cell death (73%), and DNA templated transcription (63%). Category four was also characterized with “Response to stimulus” (74% of genes), as well as System development (66.7%).

## Discussion

Here, we described a computational approach to systematically quantify and compare organismal genotype-phenotype associations across evolutionarily distant species. Our cross-species analysis of *NOA gene* orthologs revealed genes causing failure of spermatogenesis could be well studied in non-mammalian models: 89% of the *NOA genes* have an ortholog that traces back to zebrafish, fruit flies, or roundworms. Our findings indicate that the processes essential for spermatogenesis can be involved in the integument, the nervous system, the blood and immune system, the endocrine system, development, cancer progression, and more. We have shown that tissue-level gene expression, scRNA-seq, and GO annotations contain useful information for understanding whole-body genotype-phenotype patterns. We only used expression data from a selection of adult tissues. Continued investment in omics resources data will improve our abilities to interpret patterns of disease––especially important are efforts that continue to scale and integrate data from all cells of the body (Rozenblatt-Rosen et al. 2021) and across developmental time points (Coorens et al. 2025).

Computational methods for research into the whole-body biology of multicellular organisms are underdeveloped. However, the idea of systematically using full organism, full genome data for a genetic analysis platform is not new, and indeed has been a major motivation for the development of phenotype ontologies. (Washington et al. 2009) described their work as “the first effort to systematically record and computationally compare phenotype descriptions with the goal of providing a new tool for discovering genotype-phenotype relationships within and across species,” and related, foundational work was published around the same time (McGary et al. 2010; Hoehndorf et al. 2011). (Washington et al. 2009) introduced the UBERON species-neutral phenotype ontology and combined this with an Entity-Quality (EQ) methodology to perform cross-species matching of mutants with effects in the same gene or signaling pathway. (Hoehndorf et al. 2011) took a very similar approach, building species-specific ontologies into a single network, but preserving the granularity and terminology of the species-specific ontologies. (McGary et al. 2010) sidesteps the need for semantic matching of species-specific terms between two species by using a gene-first approach, and finding sets of ontology terms that appear to be under the control of the same set of genes in both species. They term these corresponding phenotype sets “phenologs.” The brilliance of this approach is to relax the assumption that gene networks in two evolutionarily distant species modulate the same phenotypes in both species, and to rely on phenotype clustering in each species to identify gene sets that continue to act as modules across species. Our work is distinct from these approaches in that (a) we use a simple ontology to describe phenotypes across five species with a single set of terms (b) we use this ontology to find new phenotype associations (“comorbidities”) for genes with established genotype-phenotype relationships in one species. We see a future role for this type of cross-species analysis as an important reference for clinical research and clinical management of disease. For instance, if reasonable support exists for a comorbidity in an accepted genetic model of a human disease, the clinician and/or clinical researcher may wish to phenotype for that comorbidity in their case(s).

This work is a pilot study for such applications, and there are several conspicuous limitations to highlight. One major limitation is that the highest resolution of the CoMo DBM gene association is at the gene level; we do not annotate or interpret the underlying alleles or perturbations for each association. In some model organisms, a well-studied gene could have dozens of alleles and perturbations with data. Many of the MCat terms are associated with Loss-of-function mutations or gene knockdown (e.g. by RNAi), but the proportion will vary across species. Missense changes and gain-of-function are still understudied, especially in vertebrates, but there is growing interest in this space (Ding et al. 2023). A long-term goal would be to label alleles in our analysis, which would improve matching of associations across species. Nearly as valuable as detailed knowledge of the alleles and phenotypes of mutant animals is knowledge of what has not been assessed or cannot be assessed. At an organismal level, it should be possible for experts to annotate which phenotype terms apply to their species. More challenging, but also valuable, would be to extend the annotation of missing data to the analysis of individual mutants/alleles. If a phenotype has not been assessed, it would be of great value to projects such as ours to be able to distinguish this status from the lack of a phenotype. Another important challenge is how to integrate and extrapolate from extensive ongoing work on mutation scanning *in vitro* with cell lines (Beltran et al. 2025; Rubin et al. 2025). A priority for simpler multicellular models like flies and worms is to understand how to bridge mutation scanning experiments, and/or the knowledge gained from them to an organismal context.

Interestingly, our analysis found that, in aggregate, NOA orthologs were not enriched for any phenotype when mutated in worm, including genitourinary defects and infertility. This is even though nearly 100% of NOA orthologs were associated with a reproductive phenotype in worms. The background rate of reproductive defects in worms is very high, due to a combination of its distinct mating system and the technical constraints of phenotyping reproduction. The MCat categories we use for defining “genitourinary defects” and “infertility” do not distinguish sex-specific effects, nor cell-type-specific effects. Roundworm hermaphrodites are 100% self-fertile (L’Hernault 2009). Since hermaphrodite mutants make sperm and oocytes, screening may not distinguish if the defect leading to sterility is in the oocyte or sperm lineage of cells. Screening may also fail to differentiate between germline and somatic defects, further complicating the phenotype classification. For instance, a paralyzed worm might be scored as “sterile” but the physiological defect is not in any specific reproductive process. We predict that NOA orthologs in worms would show a significant effect for a more refined “male infertility” phenotype category. These observations highlight the limitations of our choice to perform cross-species analysis using 19 broad phenotype categories: by lumping together traits, even in a category as distinct as “fertility,” we lose power to see specific phenotype effects. Presumably, there will be analogous effects in other organ systems.

Another consideration for this work is the looming potential of artificial intelligence (AI). In this study, we used a very focused, traditional approach to quantifying and interpreting multi-species data. We used manually generated cross-species ontologies (MCat and our reproductive ontology). We explicitly counted genotype-phenotype associations, identifying patterns across species with simple statistical testing. We further emphasized interpretability by clustering genes into a manageable number of comorbidity profiles and testing these profiles for association with gene-level metadata, including tissue expression and Gene Ontology annotations. Emerging AI-based approaches may expand the analytical space explored here. For example, graph neural network-based autoencoders, perhaps coupled with Large Language Models (LLMs), could integrate phenotype information across ontologies using textual and structural similarities and could likely extend or adapt ontologies to new species (Wang and Hu 2022). Similarly, LLM-based named entity recognition approaches can extract and normalize gene–disease relationships from unstructured biomedical text (Keloth et al. 2024). Deep learning models trained on extensive multitissue omics data and gene could help prioritize or model complex comorbidity patterns without requiring manual feature selection (Allesøe et al. 2023; Wekesa and Kimwele 2023; Schuster et al. 2024).

Looking ahead, there are clear opportunities to build on the themes presented here. On one hand, we need to accumulate data in an iterative way, focusing efforts with feedback from existing models. On the other hand, we need to design and refine computational frameworks that can learn from the data in a biologically meaningful way—ushering in a new era of AI-augmented, multi-species genetic research with direct implications for human health and disease.

## Supplementary Material

iyag038_Supplementary_Data

## Data Availability

Data is available in supplementary tables; code is available at the Conrad Lab GitHub site, https://github.com/conradlab/CoMorbidity-Database-CoMoDBM. Supplemental material available at GENETICS online.
