# Supplementary material for: Mapping whole-organism genetic comorbidities across model Species using unified ontologies
Source: Genetics. Author manuscript; Available in PMC 2026 May 11. (PMC13098149; doi:10.1093/genetics/iyag038)
Supplement: Supplementary Figure 1 [file NIHMS2158751-supplement-Supplementary_Figure_1.docx]

Supplemental Figure 1. Analysis of evolutionary conservation for various gene sets. As described in the main text, we tabulated the number of human genes with oldest orthologs in worm, fly, fish and mouse. Genes linked to azoospermia are not significantly different from random human genes in terms of their oldest ortholog distribution, but they have significantly fewer old orthologs compared to neurodegeneration genes, and significantly more old orthologs compared to immune genes. For each gene set, shown is a stacked barplot of the percentage of genes with oldest orthologs in each species.
