# Supplementary material for: Mapping whole-organism genetic comorbidities across model Species using unified ontologies
Source: Genetics. 2026 Feb 10;232(4):iyag038. doi: 10.1093/genetics/iyag038 (PMC13098149; doi:10.1093/genetics/iyag038)
Supplement: Supplementary file 1 [file iyag038_Supplementary_Data.zip › Supplementary_Table_Captions_GENETICS-2025-308907.docx]

**Supplementary Table 1**: Phenotype ontology terms for abnormalities of fertility

**Supplementary Table 2**: Descriptions of MCat categories

**Supplementary Table 3:** Mouse *NOA genes*, gene IDs and gene descriptions

**Supplementary Table 4:** *NOA gene* spermatogenesis failure categories

**Supplementary Table 5**: *NOA gene* orthologs, gene IDs, and gene descriptions *in all species*

**Supplementary Table 6**: CoMo DBM output of *NOA genes* and phenotypes in all species

**Supplementary Table 7:** Contingency tables and Fisher Test output 

**Supplementary Table 8**: COSMIC cancer data, filtered by *NOA gene* list

**Supplementary Table 9:** Human NOA orthologs annotated by disease associations in OMIM and correlation of each gene to mouse phenotype patterns. Human *NOA* genes with r>0.05 correlation are labeled as “high correlation” with *M. musculus* phenotypes. OMIM annotation summaries are the human diseases associated with each gene, grouped by a Reproductive/Non-reproductive disease/Both trait association. 

**Supplementary Table 10**: Human *NOA genes* clustered by their testis cell type expression (z-score) and Mouse *NOA genes* clustered by their phenotypes. Cluster labels “bigout_cluster” is clustering the genes by their phenotype patterns and “hista_cluster” clusters the genes by their testis cell type expression. Additional columns include HISTA expression levels and Mcat phenotype counts used for clustering. 

**Supplementary Table 11**: *NOA genes* GTEx tissue expression levels, measured by transcript per million (TPM).

**Supplementary Table 12**: Genes annotated with biological processes and molecular functions from MGI. “NA” columns indicate that a gene had been queried but fields are blank. In this case, the database may have missing data, the gene is not included in GO analysis, or the genes may not yet be characterized.
